# Autoimmune-like Drug-induced Liver Injury Caused by Atorvastatin and Demonstration of the Safety Profile of Pravastatin: A Case Report and Literature Review

**DOI:** 10.7759/cureus.7299

**Published:** 2020-03-17

**Authors:** Adeel A Khan, Salma Ahmed, Abdul Mohammed, Abdel-Naser Y Elzouki

**Affiliations:** 1 Internal Medicine, Hamad Medical Corporation, Doha, QAT; 2 Community Medicine, Hamad Medical Corporation, Doha, QAT; 3 General Medicine, Hamad Medical Corporation, Doha, QAT; 4 Medicine, Weill Cornell Medical College, Doha, QAT

**Keywords:** drug induced liver injury, atorvastatin, pravastatin, autoimmune like

## Abstract

Statin-induced liver injury is a well-recognized but rare phenomenon with hepatocellular, cholestatic, and mixed phenotypes. Most studies do not recommend regular monitoring of liver function tests (LFTs) after starting statins unless clinically indicated. We report a case of autoimmune-like atorvastatin-induced liver injury (aminotransferases > 5 times the upper limit of normal) that was detected on routine follow-up after three months in an asymptomatic patient. In addition to elevation in transaminases, the patient had weakly positive ANAs. Anti-smooth muscle antibody (ASMA) was positive in titers of 1:680. Screening for viral hepatitis A-E was negative. Other diagnostic investigations showed complete blood examination, including eosinophils, renal function tests, electrolytes, total protein, albumin, prothrombin time, activated partial thromboplastin time (aPTT), international normalized ratio (INR), serum ferritin, and iron saturation to be in the normal range. Ultrasound and computed tomography (CT) abdomen showed normal liver, gall bladder, biliary tree, and pancreas. The patient was managed as a case of autoimmune-like drug-induced liver injury (DILI) caused by atorvastatin and the medication was discontinued. LFTs returned to completely normal 30 days after the discontinuation of atorvastatin. Furthermore, switching to pravastatin for dyslipidemia management four months after stopping atorvastatin did not lead to hepatotoxicity, illustrating the safety profile of pravastatin in patients who are unable to tolerate atorvastatin.

## Introduction

Statins, also known as 3-hydroxy-3methylglutaryl coenzyme A (HMG CoA) reductase inhibitors, are the most commonly used agents for the management of dyslipidemia and cardiovascular risk reduction. Hepatotoxicity is one of its most feared side-effects. Although asymptomatic and mild elevation of aminotransferases (AST and ALT) is common, a significant elevation in these enzymes is rare and has been reported in less than 3% cases only [1-2]. Various studies have recommended against the regular monitoring of liver function tests (LFTs) after initiating statins [3-4]. We report a case of atorvastatin related autoimmune-like drug-induced liver injury (DILI) with elevation in aminotransferases > five times the upper limit of normal. It was detected on routine follow-up monitoring in an asymptomatic patient. Furthermore, the use of pravastatin after LFT normalization showed no cross-toxicity with atorvastatin.

## Case presentation

A 57-year-old female, with a history of dyslipidemia for three to four years, not controlled on dietary and lifestyle modifications, was started on atorvastatin 40 mg once daily. Baseline LFTs were normal. The patient underwent routine LFT testing and had high total and direct bilirubin (122.8 micromol/l and 85.1 micromol/l respectively), high alkaline phosphatase (435 U/l), alanine aminotransferase (3195 U/l) and aspartate aminotransferase (2999 U/l) at three months follow-up. The patient was called to report immediately to the emergency department for further investigations. The patient’s family noticed yellowish discoloration in her eyes in the last one to two days. The patient also complained of mild low-grade fever three days ago that resolved on its own but didn’t complain of chest pain, shortness of breath, sputum production, sore throat, runny nose, abdominal discomfort, itching, vomiting, rash, or change in color of urine. The patient didn’t have any history of alcohol, paracetamol, herbal medication, or any other medication intake. There was no personal or family history of autoimmune diseases or liver diseases. The patient did not have a history of allergy to any medication. On examination, the patient was afebrile, vitally stable, conscious, alert, and had mild scleral jaundice with no stigmata of chronic liver disease. Abdominal examination was unremarkable except for mild tenderness in the epigastric and right upper quadrant regions. Respiratory, cardiac, and nervous system examination was unremarkable. In addition to the above-mentioned LFTs results, other laboratory investigations showed normal complete blood examination (including normal neutrophil count and eosinophil count), renal function tests, electrolytes, total protein, albumin, prothrombin time, aPTT, INR, serum ferritin, and iron saturation. Urine dipstick was normal. Except for borderline triglyceride of 2.2 mmol/l (normal <1.7 mmol/l), the lipid profile was within the desirable range. Screening for viral hepatitis A, B, C, and E was negative. She had a weakly positive ANA and positive anti-smooth muscle antibody (ASMA) (1:640). Anti-liver-kidney microsomes, anti-mitochondrial antibody, and screen for connective tissue diseases were negative. Serum immunoglobulin G (IgG) levels were within the normal range (1210 mg/dl). Ultrasound and CT abdomen showed normal liver, gall bladder, biliary tree, and pancreas (Table 1).

**Table 1:** Baseline characteristics

Age (years)	57
Gender	Female
Peak Total Bilirubin (3.4-20.5 micromol/L)	90.1
Peak Direct bilirubin (0.0-8.6 micromol/L)	60.3
Peak ALT (Alanine Aminotransferase, Normal = 0-55 U/L)	3756
Peak AST (Aspartate Aminotransferase, Normal = 5-34 U/L)	3172
Peak Alkaline Phosphatase (40-150 U/L)	397
Viral Hepatitis Serology	Negative
Time of Onset of Elevation in LFTs (Liver Function Tests)	Within 3 months
Time of Return of LFTs (Liver Function Tests) to Normal	Within 30 days
RUCAM (Roussel-Uclaf Causality Assessment Method) Score	9 (highly probable)

The patient was advised liver biopsy to rule out autoimmune hepatitis based on positive autoimmune antibody results but refused and preferred to monitor LFTs on a regular basis. The patient was managed as a case of atorvastatin-induced autoimmune-like DILI. Atorvastatin was discontinued and the patient was followed up in the outpatient clinic with regular monitoring of her LFTs. Twenty-four days after stopping atorvastatin, LFTs improved, indicating drug-induced etiology of elevation in liver enzymes. Total bilirubin became 32.1 micromol/l, alkaline phosphatase normalized at 121 U/l, ALT 211 U/l, and AST 88 U/l. Aminotransferases returned to normal after one month. After stopping atorvastatin, serum ASMA levels decreased to 1:80 in titers.

Follow-up of the patient’s lipid profile three months after stopping atorvastatin showed very high total cholesterol (7.2 mmol/l) and LDL (5.58 mmol/l) levels. Pravastatin was introduced and follow up with LFTs was done for 18 months, which showed no elevation in aminotransferases on pravastatin (Table 2).

**Table 2:** Liver function before and after stopping atorvastatin

	Baseline	At diagnosis	24 days after stopping atorvastatin	30 days after stopping atorvastatin	14 days after starting pravastatin	6 months after starting pravastatin	10 months after starting pravastatin	18 months after starting pravastatin
Total Bilirubin (3.4-20.5 micromol/L)	7.5	90.1	32.1	11	8.4	10	7	6
Direct Bilirubin (0.0-8.6 micromol/L)	-	60.3	21.6	-	-	-	-	
ALT (Alanine Aminotransferase, Normal = 0-55 U/L)	26	3756	211	17	18	22	22	18
AST (Aspartate Aminotransferase, Normal = 5-34 U/L)	23	3172	88	25	19	23	23	18
Alkaline Phosphatase (40-150 U/L)	95	397	121	74	77	71	73	71

This illustrates the safety profile of pravastatin in patients who require statins for high LDL management but develop hepatic side-effects on atorvastatin.

## Discussion

Drug-induced liver injury is categorized into three phenotypes based on initial R-value; hepatocellular (R-value > 5), cholestatic (R < 2), and mixed (R > 2 but < 5). R-value is calculated by the ratio of serum ALT to serum alkaline phosphatase. Our patient had initial R-value > 5 that attributed her to the hepatocellular group [5]. The Roussel-Uclaf causality assessment method (RUCAM) score is used to estimate the probability that hepatotoxicity has been caused by the drug based on the type of liver injury, timing of onset, assessment of risk factors, course of the reaction, exclusion of other causes, and response to re-administration. A score of 0 or less excludes drug-related causes. A score of 1-2 Is characterized as “unlikely,” 3-5 as “possible,” 6-8 as “probable,” while 9 and above is labeled as “highly probable” drug-related liver injury [6]. Based on our patient characteristics, the patient was found to be in the “highly probable” category. A subset of DILI named “autoimmune-like DILI” has been identified and is difficult to differentiate from “autoimmune hepatitis (AIH)” due to the considerable overlap of clinical and laboratory features [7]. In the event of autoimmune-like DILI, ALT and AST return to normal soon after the discontinuation of the offending agent in most cases without the requirement for immunosuppressants. Furthermore, if AIH is misdiagnosed as immune-mediated DILI and immunosuppressants are used to manage it, there will be a relapse of AIH on cessation of immunosuppressants but DILI almost never relapses after the stoppage of the offending agent [8]. Liver biopsy is not mandatory to make a diagnosis of DILI and is mainly used to rule out other causes [9]. Russo et al. reported 12 (out of a total of 22) cases of statin-induced liver injury of hepatocellular phenotype, out of which six had autoimmune-like DILI with 67% of the cases having ANA and ASMA levels > 1:40 [5]. In our patient, ANAs were weakly positive and so was ASMA with 1:640 titers (that later decreased to 1:80 titers after the discontinuation of atorvastatin) showing autoimmune-like DILI.

The latency period of elevation in aminotransferases after statin initiation is variable, with most of them occurring within 12 weeks [10]. However, latency periods of as long as one year have been observed as well [5]. NICE guidelines recommend baseline LFTs testing before starting statins, then within three months and at 12 months followed by testing only if the patient develops any symptoms related to statins side-effects [11]. American College of Cardiovascular Administrators (ACCA)/American Hospital Association (AHA) guidelines recommend measuring baseline LFTs followed by testing only if clinically indicated [12]. Based on our experience with this case, we recommend the routine follow-up testing of LFTs in asymptomatic patients at least in the initial few months when the risk of hepatotoxicity is highest.

Restarting statin after an episode of liver toxicity is discouraged unless clinically necessary and no other suitable alternative is available [9]. There is limited data on the risk of cross-toxicity between different statins in cases of statin-induced liver injury. However, Liu et al. reported two cases of atorvastatin-induced liver injury who were switched to pravastatin after the return of transaminases to baseline. In both cases, no significant elevation in transaminases from baseline was observed on follow-up [13]. In our patient as well, the use of pravastatin for dyslipidemia management was found to be safe and no derangement of LFTs was observed after 18 months of follow-up, endorsing the possibility that pravastatin has little risk of cross-toxicity with atorvastatin and can be safely instituted if clinically indicated in patients who had DILI with atorvastatin.

## Conclusions

Statins-induced liver injury can present in the hepatocellular, cholestatic, or mixed pattern. A subset of liver injury caused by statins called "autoimmune-like drug-induced liver injury" can mimic autoimmune hepatitis. It can be differentiated from autoimmune hepatitis by the normalization of aspartate aminotransferase and alanine aminotransferase levels after the withdrawal of statins therapy. As most cases of statins-induced liver injuries occur early during the initiation of statins therapy, liver function tests should be routinely monitored in the initial few months after starting statins. Furthermore, if a patient develops liver injury due to atorvastatin use, pravastatin has shown a reduced risk of hepatotoxicity in such patients and may be safely used after the normalization of liver enzymes if clinically indicated.
